# Clear cell carcinoma arising from abdominal wall endometriosis—a report on two cases and literature review

**DOI:** 10.1186/s12957-022-02553-x

**Published:** 2022-03-15

**Authors:** Vishal Bahall, Lance De Barry, Arlene Rampersad

**Affiliations:** 1 Department of Obstetrics and Gynaecology, San Fernando General Hospital, South-West Regional Health Authority, San Fernando, Trinidad and Tobago; 2 Department of Obstetrics and Gynaecology, South-West Regional Health Authority, San Fernando, Trinidad and Tobago; 3 Department of Pathology, San Fernando General Hospital, South-West Regional Health Authority, San Fernando, Trinidad and Tobago

## Abstract

**Background:**

Malignant transformation of abdominal wall endometriosis is extremely rare. Clear cell carcinoma and endometrioid carcinoma are the two most prevalent histological subtypes of malignant endometriosis. To date, approximately, thirty cases of clear cell carcinoma arising from abdominal wall endometriosis have been described worldwide.

**Case presentation:**

We report two cases of clear cell carcinoma developing postoperatively in the anterior abdominal wall in women with a history of extensive endometriosis. Histopathology of the resected abdominal wall tumor demonstrated benign endometriosis contiguous with features of clear cell carcinoma. These histological features satisfied Sampson’s criteria which are required for diagnosing malignant endometriosis. Both patients were successfully managed with platinum-based adjuvant chemotherapy following cytoreductive surgery.

**Conclusion:**

Clear cell carcinoma arising from the abdominal wall endometriosis is a rare, highly aggressive cancer with a propensity to recur or metastasize. Due to the limited publications on this clinical entity, there are no clearly established protocols regarding adjuvant treatment, and an evaluation of prognostic factors is lacking. Clinicians must have a high index of suspicion for malignant endometriosis of the abdominal wall, particularly in patients with an abdominal wall mass, prior abdominal surgery, and long-standing endometriosis. By presenting our case, we expect to raise awareness and study of this rare endometriosis-related neoplasm.

## Introduction

The abdominal wall is a common site of extraperitoneal endometriosis, and it typically occurs following the cesarean section, hysterectomy, laparoscopic trocar placement, episiotomy, and hernia repair [1]. Although the complications of endometriosis are diverse, malignant transformation of endometriotic lesions is rare and carries a 0.7–1.0% incidence rate [2].

Sampson et al. defined three criteria for diagnosing an endometriosis-related neoplasm: (1) evidence of endometriosis in proximity to the tumor, (2) absence of another primary site tumor, and (3) histological evidence consistent with an endometrial origin [3]. Clear cell carcinoma is the most common histologic subtype of malignant endometriosis, followed by endometrioid cancer [4].

Clear cell carcinoma arising from the abdominal wall endometriosis presents as a slow-growing mass adjacent to a scar from previous surgery and cyclical abdominal pain [5]. These symptoms may be easily mistaken for benign conditions such as a hernia, abscess, lipoma, or hematoma [6]. The management of clear cell carcinoma of the abdominal wall necessitates cyto reductive surgery with considerations for adjuvant platinum-based chemotherapy [5]. In general, the prognosis of this condition is poor as disease recurrence or progression is common.

Herein, we report two cases of clear cell carcinoma of the abdominal wall which developed postoperatively in patients with a history of long-standing endometriosis. Both patients were managed with surgical debulking and platinum-based chemotherapy. One patient developed recurrence in the inguinal and pelvic lymph nodes which necessitated lymph node dissection and further chemotherapy.

## Case description

### Case 1

A 46-year-old nulliparous female presented to us with a 4-month history of a progressively enlarging suprapubic abdominal mass associated with worsening pelvic pain. She denied experiencing symptoms of fever, weight loss, vaginal bleeding, discharge, and urinary or gastrointestinal complaints. She had a history of endometriosis for 15 years, and her past surgical history is significant for laparoscopic ovarian drilling 10 years prior. The patient had no personal or family history of malignancy.

On clinical examination, a *10cm x 8cm* suprapubic abdominal mass was noted with extension to the mons pubis. The mass was non-tender, immobile, and firmly attached to the abdominal wall. The overlying skin appeared erythematous with areas of necrosis secondary to the pressure effects of the mass. The uterus was also enlarged and extended to the umbilicus. There were also no clinical signs of gross ascites.

**Fig. 1:**
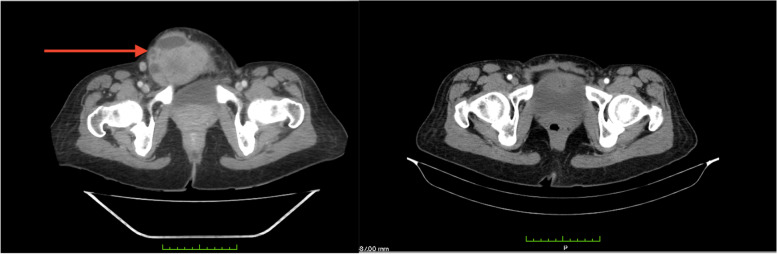
Axial CT images of the abdomen and pelvis. **A** Before resection. **B** After surgery (resection of the abdominal wall mass, hysterectomy, bilateral salpingo-oophorectomy, and closure with polypropylene mesh

Blood investigations revealed a hemoglobin concentration of *8.7 g/dl* and elevated tumor markers: CA125- *1200 U/ml*, CA15-3- *425.8 U/ml*, CA19-9- *6610.8 U/ml*, and a CEA- *10.2 U/ml*. An HIV rapid test was negative. Computed tomography (CT) scan of the chest, abdomen, and pelvis (Fig. 1) demonstrated normal appearances of the intra-abdominal viscera and an enlarged uterus measuring *16.5cm x 10.1cm x 11.5cm* with multiple intramural and subserosal leiomyomas. A low anterior abdominal wall mass arising from the rectus abdominis muscle was also noted. The mass measured *10.8cm x 7.8cm x 8.*5cm and crossed the midline, extending to the left iliac fossa. Prominent pelvic lymph nodes were also noted. Additionally, there were bilateral ovarian cysts, right and left measuring *6.1cm x 6.0cm* and *4.4cm x 3.2cm*, respectively.

An ultrasound-guided biopsy of the mass exhibited papillary structures while immunohistochemistry demonstrated positive stains for CK7, CK20, ER, p53, and a negative stain for WT-1. These features were suggestive of adenocarcinoma, likely related to the female genital tract. Furthermore, immunotyping was not typical of a serious type or primary peritoneal carcinoma.

The patient subsequently underwent an exploratory laparotomy, resection of the abdominal wall mass, total hysterectomy, bilateral salpingo-oophorectomy, pelvic lymph node sampling, and reconstruction of the rectus sheath with a polypropylene mesh. Intraoperatively, endometriotic deposits were noted throughout the pelvic cavity. The intra-abdominal viscera appeared unremarkable, and there was no evidence of ascites.

Histopathology of the abdominal wall mass indicated clear cell carcinoma associated with a solid, acinar pattern, and a 1-mm clear margin. The tumor cells exhibited marked nuclear atypia and clear cytoplasm. Intraglandular necrosis admixed with eosinophilic material was noted. The tumor occurred within a cyst-like structure lined by aggregates of siderophages and hemosiderin. This suggested an origin from a focus of “burnt out” endometriosis. Endometriotic deposits were found elsewhere in the excision sample including both fallopian tubes and ovaries. Lymph nodes were negative for metastatic involvement.

The patient’s case was discussed at the multi-disciplinary team (MDT) meeting, and she was referred to the Medical Oncology team for adjuvant chemotherapy. The chemotherapy regime consisted of six cycles of paclitaxel (135mg/m^2^) and carboplatin (720mg (AUC-6)) given every 21 days. During treatment, a monthly evaluation of a CA-125 level was done followed by every 3 months after treatment was completed. CA-125 levels steadily decreased during treatment and were within normal limits upon completion of treatment. The patient is now 32 months since her treatment concluded and is currently followed up in both Gynae-Oncology and Medical Oncology clinics with no evidence of recurrence.

### Case 2

A 57-year-old female presented with a 1-year history of an abdominal wall mass at the right margin of a previous Pfannensteil scar, and a 4-day history of increasing abdominal pain. The patient denied experiencing nausea, vomiting, fever, weight loss, and urinary or bowel symptoms. She is a known diabetic and hypertensive with a 20-year history of endometriosis. Her past surgical history is significant for an umbilical hernia repair 26 years prior, two cesarean sections 27 and 29 years ago, and a total abdominal hysterectomy with ovarian conservation secondary to symptomatic uterine leiomyomas 15 years prior. She had no personal or family history of malignancy.

**Fig. 2:**
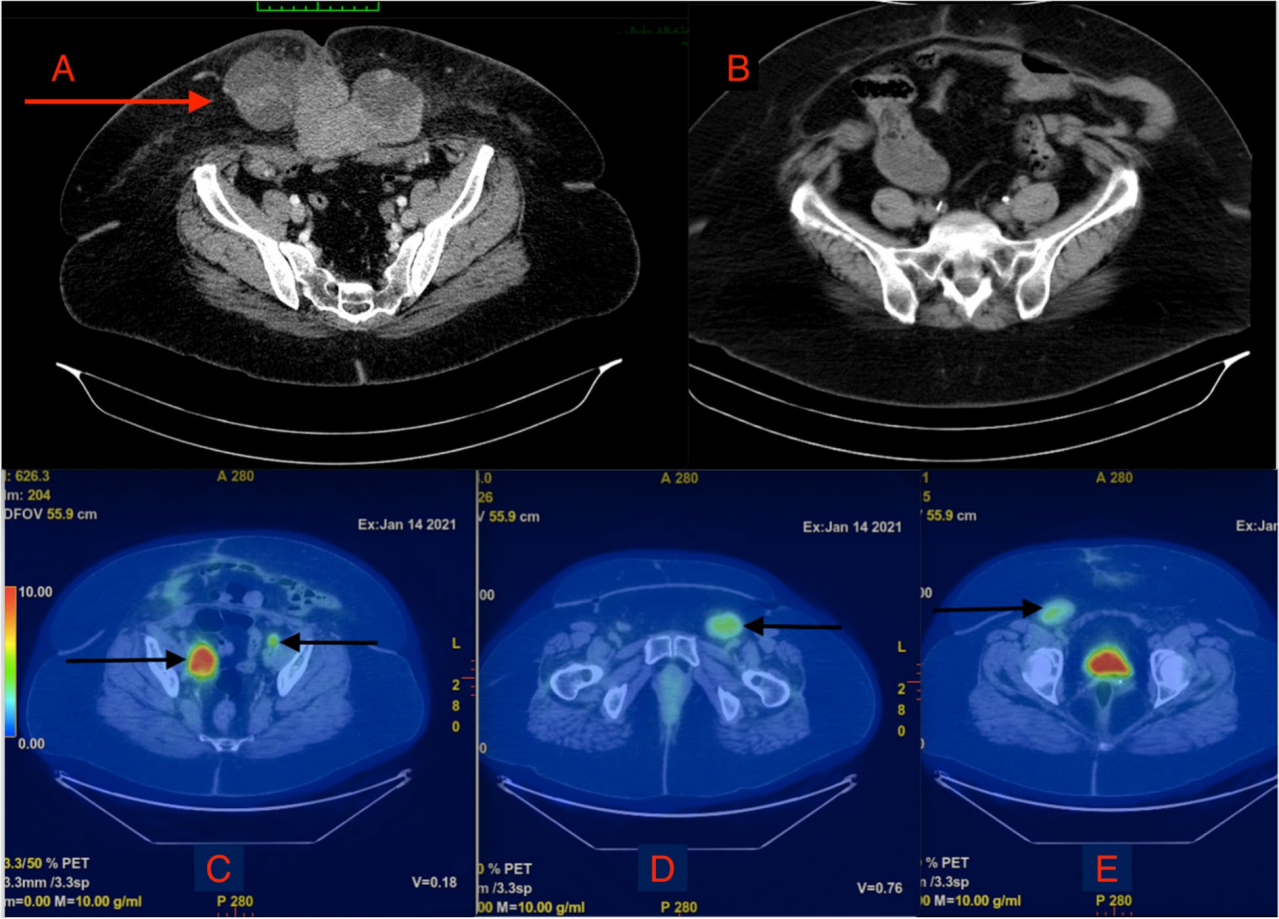
Axial CT images of the abdomen and pelvis. **A** Before resection and **B** after surgery—laparotomy with the removal of the abdominal wall mass, lymph nodes, and closure with polypropylene mesh. PET scan images (**C**, **D**, **E**) demonstrating recurrent carcinoma in the pelvic, left inguinofemoral, and right inguinofemoral nodes, respectively

On clinical examination, a *15cm x 15cm* firm, immobile, irreducible, and non-tender mass was noted in the right lower quadrant of the abdomen with areas of superficial skin necrosis. A CT scan of the abdomen and pelvis demonstrated a heterogenous enhancing mass in the anterior abdominal wall measuring *15cm x 8cm x 13.8cm* with involvement of the right rectus abdominis muscle (Fig. 2). Partial protrusion into the abdominal cavity was noted. Anterior abdominal wall lymphadenopathy was noted; however, there was no evidence of lymphadenopathy elsewhere. The intra-abdominal viscera appeared unremarkable, and there was no evidence of a hernia or ascites.

**Fig. 3:**
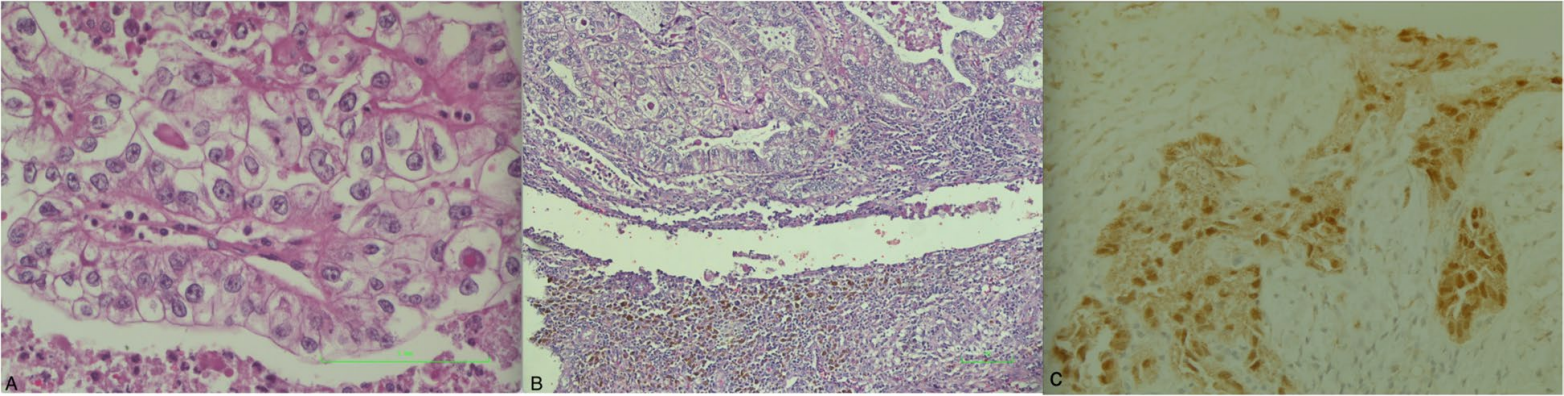
Histopathology demonstrating. **A** Clear cell carcinoma with acini lined by markedly pleomorphic cells with abundant clear cytoplasm. **B** Aggregates of siderophages and hemosiderin bordering a clear cell carcinoma arising from the pelvic wall and **C** PAX 8 positivity

An ultrasound-guided biopsy of the abdominal mass was performed. Immunohistochemistry showed a tumor that expressed weak positivity for CK7 and strong positivity for PAX8 (Fig. 3A, C). Tumor cells were ER, WT1, and CK20 negative. This immunohistochemistry profile concluded moderately differentiated metastatic adenocarcinoma with a primary origin from the reproductive tract or kidneys.

At the MDT meeting, a decision was made for surgical resection of the abdominal wall mass with accompanying lymph nodes, abdominal wall reconstruction, and bilateral salpingo-oophorectomy. Intraoperatively, a *15cm x 13cm* irregular, right-sided abdominal wall mass involving the right rectus abdominis muscle and rectus sheath was removed. Post-status hysterectomy changes were observed, and endometriotic deposits were noted throughout the pelvic cavity. A total of eight abdominal wall lymph nodes were excised. There were no ascites, and the intra-abdominal organs appeared unremarkable. The abdominal wall was repaired using a polypropylene mesh.

Histopathology (Fig. 3B) indicated that the abdominal wall mass was a clear cell carcinoma with a prominent tubulopapillary pattern. The papillae had hyalinized eosinophilic cores and the tumor cells displayed mild to moderate nuclear atypia and clear cytoplasm. Among these malignant cells, foci of endometriotic glands and stroma were noted along with hemosiderin-laden macrophages. Immunohistochemistry indicated strong diffuse expression of PAX-8. Eight abdominal wall lymph nodes were positive for metastasis. The patient was subsequently referred to the Medical Oncology unit for adjuvant chemotherapy.

This patient was placed on six cycles of chemotherapy inclusive of paclitaxel (135mg/m^2^) and carboplatin (583mg (AUC-5)) repeated every 21 days after MDT discussion. The patient was also monitored with monthly CA-125 levels during adjuvant treatment.

However, after the completion of six cycles of chemotherapy, CT imaging demonstrated localized disease in the pelvic and inguinal lymph nodes. These lesions were confirmed to be new areas of involvement when compared to pre-surgical imaging. A decision was made at the MDT meeting to have a whole-body FDG (fluorodeoxyglucose) positive emission tomography (PET) scan followed by potential surgical intervention.

A whole-body FDG PET scan (Fig. 2) revealed increased sizes of new bilateral para-iliac, right femoral, and left inguinal FDG avid lymph nodes. The most prevalent FDG avid lymph node was noted in the right para-iliac region and demonstrated a standardized uptake value (SUV) max of 10.9. This indicated a likely recurrence of clear cell carcinoma. FDG avid adenopathy was not observed elsewhere in the body, and there was no evidence of hypermetabolic uptake in the anterior abdominal wall to suggest progressive or residual disease.

Based on the PET scan findings, the patient underwent a laparotomy with removal of bilateral pelvic and inguinal lymph nodes. Histopathology confirmed recurrent clear cell carcinoma. A decision was made for the continuation of adjuvant chemotherapy as there was no evidence of disease in previously resected areas using this treatment regime. Currently, this patient is undergoing further adjuvant chemotherapy treatment with paclitaxel and carboplatin. She is receiving follow-up care in the Gynae-Oncology and Medical Oncology clinic.

## Discussion

Clear cell carcinoma arising from abdominal wall endometriosis is a highly aggressive, rare cancer with a propensity to recur or metastasize [7]. Approximately thirty cases have been described worldwide since it was first reported by Sampson in 1925 [8]. In general, malignant transformation of endometriosis is highly unusual and approximately 0.7–1.0% of women diagnosed with endometriosis develop an endometriosis-related neoplasm [2]. According to Taburiaux et al., malignant transformation of the abdominal wall endometriosis develops 21 years after initial uterine surgery, while the average age at diagnosis is 47 years [9].

Malignant endometriosis typically affects the ovary while, extra-gonadal malignant endometriosis may involve the colon, rectovaginal septum, and vaginal walls [10, 11]. Rarely, the abdominal wall is implicated. The most common histologic subtype of extra-gonadal malignant endometriosis is endometrioid carcinoma (69.1%) followed by sarcoma (25%) and clear cell carcinoma (4.5%) [5]. However, when malignant endometriosis involves the abdominal wall, the predominant histological subtype observed is clear cell carcinoma (66%) followed by endometrioid carcinoma (24%) [5]. Clear cell carcinoma arising from abdominal wall endometriosis is similar to clear cell carcinoma of the endometrium, ovary, vagina, and cervix. These cancers are deeply invasive and carry a poor prognosis [12].

The pathogenesis of malignant transformation of endometriosis remains unelucidated; however, certain studies implicate oxidative stress according to a two-step process in endometriotic carcinogenesis [13]. Firstly, the fluid in endometriotic cysts contains elevated levels of iron which may form reactive oxygen species and promote oxidative stress [14]. Oxidative stress may lead to epigenetic alterations and aberrant DNA methylation which predispose susceptible cell populations to malignant transformation [15]. In the second step, cancer progression occurs due to antioxidant production which prolongs the survival of affected cell populations, thus promoting carcinogenesis [13, 14].

Several risk factors have been described for the malignant transformation of endometriosis. These include hyperestrogenism, endometriosis diagnosed at an early age, women with long-standing endometriosis, ovarian endometriomas, and endometriosis associated with infertility [16]. Additionally, carcinogens like dioxin, and genetic anomalies involving the loss of heterozygosity on chromosome 4, may be implicated [10].

Clear cell carcinoma of the abdominal wall presents as an abdominal mass that develops adjacent to a previous surgical scar [5, 17]. The tumor often involves the rectus abdominis muscle and may protrude into the abdominal cavity as noted in the second case described [18]. Abdominal pain is commonly reported and may occur as the tumor invades nearby anatomical structures. Additionally, premenopausal women may experience cyclical abdominal pain which reflects the presence of hormonally active endometriotic foci [19]. A diagnostic dilemma often arises in the evaluation of women with clear cell carcinoma of the abdominal wall. This clinical entity may be mistaken for various benign causes of an abdominal wall mass such as an abscess, hernia, lipoma, hematoma, or lymphadenopathy [6]. Hence, for the generalist, a thorough evaluation should entail a detailed gynecological history and examination, imaging studies, and biopsy as the clinical suspicion for an endometriosis-related neoplasm may be low [17].

Currently, there are no pathognomonic biochemical markers for malignant endometriosis. The cancer antigen, CA-125, is a non-specific biomarker elevated in both ovarian malignancy and advanced endometriosis [5]. Thus, a considerable increase in CA-125 concentrations may increase the suspicion of malignancy [20]. Imaging modalities such as CT and magnetic resonance imaging (MRI) of the chest, abdomen, and pelvis are important to delineate the extent of disease, invasion into adjacent structures, and metastasis; exclude benign causes of an abdominal wall mass; and plan surgical intervention [8].

The diagnosis of clear cell carcinoma is confirmed on histopathology. Histologically, clear cell carcinoma exhibits papillary, tubulocystic, or solid patterns [12]. Other common histologic findings include intraluminal mucin, intracytoplasmic vacuoles, eosinophilic hyaline mucin, and stromal hyalinization [12]. Moreover, immunohistochemistry may demonstrate positive stains for HNF-1B, Napsin-A, AMACR, CK-7, and p53 [21]. Of significance, in 1925, Sampson defined three criteria for confirming the diagnosis of neoplastic transformation of endometriosis: (1) evidence of endometriosis in proximity to the tumor, (2) absence of another primary site tumor, and (3) histological evidence consistent with an endometrial origin [3]. Scott et al. added a fourth criterion in 1953: morphological demonstration of benign endometriosis contiguous with malignant tissue [22]. In both cases described, histopathology fulfilled Sampson’s three criteria and Scott’s fourth criterion, thus confirming an endometriosis-related clear cell carcinoma.

The management of clear cell carcinoma arising from abdominal wall endometriosis consists of cytoreductive surgery with considerations for adjuvant chemotherapy [5]. Surgical intervention necessitates wide excision of the abdominal wall tumor to achieve healthy margins, hysterectomy, bilateral salpingo-oophorectomy, and abdominal wall reconstruction [5, 17]. According to Ferrandina et al., a bilateral salpingo-oophorectomy and endometrial biopsy should be performed to exclude other primary tumor sites [23]. Lymph node dissection is considered when pre-operative imaging demonstrates suspicious nodes or nodal involvement [24]. A prosthetic mesh or in some cases, a pedicle-skin-muscle flap, is used for abdominal wall repair and reinforcement depending on the extent of surgical resection [23].

Due to the limited publications on this clinical entity, there are no clearly established protocols regarding adjuvant treatment [7]. However, clear cell carcinoma arising from abdominal wall endometriosis appears to respond to adjuvant platinum-based chemotherapy [17]. The combination of carboplatin (AUC 5-6) and paclitaxel (175mg/m^2^) used for six cycles demonstrates efficacy in treatment, and this combination is better tolerated and less toxic compared to the use of doxorubicin, carboplatin, and paclitaxel triple therapy [8, 25, 26]. Despite the use of adjuvant chemotherapy, disease recurrence or progression may still occur, as noted in our study [7]. Adjuvant radiotherapy may be considered, particularly in cases with a suboptimal response to chemotherapy [17]. However, reports indicating successful outcomes with radiotherapy are scarce, and there are no standardized treatment protocols available.

Clear cell carcinoma of the abdominal wall is associated with a poor prognosis [12]. Patients often encounter disease recurrence or progression. Lymph node metastasis is common and particularly involves the inguinal lymph nodes since the superficial abdominal wall lymphatics drain towards the superficial inguinal nodes [27]. Due to the rarity of cases, an evaluation of prognostic factors is also challenging. According to Taburiaux, the median survival time is approximately 30 months [9]. Patients should be followed-up indefinitely and this should involve regular pelvic and rectal examinations, regional lymph node examinations, serum CA-125 measurements, and imaging for suspected recurrence.

In conclusion, clear cell carcinoma arising from the malignant transformation of abdominal wall endometriosis is a rare and aggressive cancer associated with a poor prognosis. This condition may be easily mistaken for many benign causes of an abdominal wall mass. Hence, clinicians must be suspicious of malignancy in patients with a history of abdominal surgery and long-standing endometriosis. Until treatment protocols are established, cytoreductive surgery with abdominal reconstruction and adjuvant chemotherapy utilizing platinum-based compounds appear to improve outcomes. Adjuvant radiotherapy should be considered in select cases with a suboptimal response to adjuvant chemotherapy.

## Data Availability

Not applicable.
